# Basal-like breast cancer with low TGFβ and high TNFα pathway activity is rich in activated memory CD4 T cells and has a good prognosis

**DOI:** 10.7150/ijbs.56128

**Published:** 2021-01-30

**Authors:** Dingxie Liu, Jaydutt Vadgama, Yong Wu

**Affiliations:** 1 Bluewater Biotech LLC, New Providence, NJ, USA.; 2 Division of Cancer Research and Training, Department of Internal Medicine, Charles Drew University of Medicine and Science, David Geffen UCLA School of Medicine and UCLA Jonsson Comprehensive Cancer Center, Los Angeles, CA 90095, USA.

**Keywords:** Basal-like breast cancer, immunity-related pathways, chemotherapy, prognosis

## Abstract

Basal-like breast cancer (BLBC) is a type of high-grade invasive breast cancer with high risk of recurrence, metastases, and poor survival. Immune activation in BLBC is a key factor that influences both cancer progression and therapeutic response, although its molecular mechanisms are not well clarified. In this study, we examined five cancer immunity-related pathways (IFNα, IFNγ, STAT3, TGFβ and TNFα) in four large independent breast cancer cohorts (*n* = 6,381) and their associations with the prognosis of breast cancer subtypes. Activities of the 5 pathways were calculated based on corresponding pathway signatures and associations between pathways and clinical outcomes were examined by survival analysis. Among the five PAM50-based subtypes, BLBC had the highest IFNα, IFNγ, TNFα pathway activities, and the lowest TGFβ activity. The IFNα, IFNγ, TNFα pathway activities were negatively correlated with BLBC recurrence. In contrast, positive association and no association with BLBC recurrence were observed for TGFβ and STAT3 pathways, respectively. TNFα/TGFβ pathway combination improved the prediction of recurrence and chemotherapy response of BLBCs. Immune cell subset analysis in BLBC showed that M0, M1 and M2 macrophage levels were associated with either TNFα or TGFβ pathways, whereas the level of activated memory CD4 T cells were associated with both pathways. Moreover, this T cell subset was most abundant in BLBCs with low TGFβ and high TNFα pathway activities. These results suggested that cooperation of TNFα and TGFβ signaling may be involved in the regulation of memory T cells and anti-cancer immunity in BLBCs. Our data also demonstrate that TNFα/TGFβ pathway combination may represent a better biomarker for BLBC prognosis and clinical management.

## Introduction

Breast cancer is the most common malignant tumor in women and a heterogeneous disease that has been demonstrated to be divided into five major biologically distinct intrinsic subtypes based on gene expression profiling, i.e. luminal A, luminal B, human epidermal growth factor receptor-2 (HER2) overexpressing, basal-like, and normal-like 1-3. Of the five intrinsic subtypes, basal-like breast cancer (BLBC) is of particular concern since it does not express estrogen receptor (ER), progesterone receptor (PR), and HER2, and hence does not benefit from anti-estrogenic therapy or trastuzumab 4. BLBC is likely to be a high-grade invasive tumor with active mitosis and is related to younger patient age 5, 6.

Recently, whole genome analysis has been supplemented to the equipment library of experimental techniques, which provides a new molecular classification for breast cancer and promotes the development of many prognostic multigene analyses 7-13. The initial gene expression profile analysis showed that BLBCs and HER2 overexpression subtypes are associated with exceptionally poor prognosis 14-16. In contrast, patients with Luminal A cancers have a good prognosis 2, 3. Nevertheless, after careful examination, these studies further indicate that the prognosis of BLBC patients is highly time-dependent. Some patients with BLBCs have a particularly low survival rate in the first 3-5 years after diagnosis, nonetheless for others, their mortality rate decreases, so the survival rate of these patients is better than that of patients with luminal (ER1) tumors at 10 years after diagnosis 17-20. This indicates that patients with BLBCs can be divided into two clinically distinct groups: those who are likely to relapse and die of their disease within the first three to five years of diagnosis, and those who are expected to show good long-term survival. Therefore, the indexes that can be used to accurately predict the prognosis of this kind of patients are particularly important and have clinical significance.

Although there are a variety of multi-gene signatures that can predict the prognosis of patients with breast cancer, their prognostic value seems to come mainly from their ability to measure the expression of proliferation-related genes 21, 22. Since BLBC is usually highly proliferative, the existing prognostic signatures fail to identify BLBC subsets with a favorable prognosis 23. Some recent studies have focused on determining multi-gene predictors for the prognosis of triple-negative (ER-, PR-, HER2-) and hormone receptor negative breast cancers 22-27. Nevertheless, a reliable method to distinguish between good and poor prognosis of BLBC has not been developed. For this purpose, we have begun to optimize such a method, and here we report the identification of an immunological pathway signature combination, which is related to the prognosis of patients with BLBCs.

Although genetic/epigenetic alterations are key pathogenic events, the immunological signaling plays an important role in promoting progression and metastasis of BLBCs. Previously, we examined multiple cancer-related pathway signatures in colon and breast cancers 28-30. Five of these signatures, including IFNα, IFNγ, STAT3, TGFβ and TNFα pathway signatures, are cancer immunity-related. In this study, we analyzed the associations of these signatures with the prognosis of PAM50-based intrinsic subtypes of breast cancers, with an emphasis on BLBC.

## Methods

### Microarray datasets of breast cancer

In our previous study 29-31, we tested 42 Affymetrix U133 microarray datasets, which were again used to examine the correlation between immunological pathway signatures and basal-like breast cancer. In the 42 microarray datasets, 15 datasets contain patients' neoadjuvant response information, and 30 data sets contain cancer recurrence information (3 data sets contain both information). We described in detail the normalization of the raw array data, the elimination of batch effects, and the clinical characteristics of patients in these datasets in our previous work 29, 31.

We previously merged the 42 microarray datasets into 4 cohorts 29-31. Cohort 1 was used here to examine the association of pathway signatures with neoadjuvant response of BC because it was merged from 15 datasets with only patient neoadjuvant response information (i.e. MDA133, GSE16446, GSE18728, GSE18864, GSE20194, GSE20271, GSE25055, GSE25065, GSE26639, GSE32646, GSE37946, GSE41998, GSE42822, GSE50948, and GSE66305). The other 3 cohorts were used to examine the associations of pathway signatures with BC relapse. Cohort 2 contains 16 datasets (GSE11121, GSE12276, GSE2034, GSE17705, GSE2603, GSE20685, GSE2990, GSE26971, GSE3494, GSE45255, GSE58812, GSE6532, GSE65194, GSE7390, GSE9195, GSE88770) in which metastasis date is available. Cohort 3 comprises 8 datasets (GSE12093, GSE20711, GSE21653, GSE31519, GSE42568, GSE1456, GSE7378, GSE71258) in which only relapse date information is available. The remaining 6 datasets with RFS or DMFS information (GSE16391, GSE16446, GSE17907, GSE19615, GSE25055, and GSE25065) were merged as the Cohort 4 because the follow-up time of these datasets (about 5 years) was much shorter than that of cohorts 2 and 3. The batch effect in the four merged cohorts was eliminated using the R Combat function, as we showed previously 29. The disease-free survival time in cohort 2 and cohort 3 was censored at 8 years, while it was censored at 5 years in cohort 4.

### Pathway signature generation and pathway activity prediction using BinReg

Using the BinReg approach to generate pathway signatures and predict pathway activities of individual samples has been described in detail before 32, 33. Briefly, the gene expression patterns of two groups of samples (with one pathway being 'on' and 'off' in the two groups respectively) are analyzed to identify the pathway-specific informative genes (signature genes). Principal components are then used to compute weights for each of signature genes, such that the weighted average of expression levels show a clear ability to distinguish the pathway “on” and “off” groups. By applying binary regression on the principal components to the gene expression dataset of a test sample, a probability score of pathway activity for that sample will be produced.

The signatures for 11 pathways analyzed in this study, except for BRAF/MEK and AR pathways, were previously reported by Gatza et al 32, 33. According to the authors' suggestion, MAS5.0 normalized gene expression data was used to predict the activity of p53, STAT3, and TNFα pathways, while RMA normalized data was used for Wnt/β-catenin (BCAT), EGFR, IFNα, IFNγ, PI3K and TGFβ pathways. AR signature was generated in our previous study 29, while BRAF/MEK was generated in the present study. Both of these two signatures were based on RMA-normalized expression data.

IFNα, IFNγ, TGFβ and TNFα pathway signatures were generated and validated using gene expression data from cells treated with or without IFNα, IFNγ, TGFβ and TNFα, respectively 33. We previously generated a gene signature for mutant BRAF signaling 34. Since *BRAF* mutation is rare in breast cancer, here we generated a BRAF/MEK signature based on the cells treated with mock or MEK-specific inhibitor (Supplementary Fig. **S1**). The conditions for the BRAF/MEK pathway signatures are listed in Supplementary Table **S1**.

### Score calculation of two pathway signature combination

The pathway signature scores were first Z-sore transformed. The scores for combination of TGFβ with IFNα, IFNγ, and TNFα were calculated by subtracting the TGFβ signature score from IFNα, IFNγ, and TNFα signature scores respectively (e.g., score of TNFα/TGFβ combination = TNFα signature score - TGFβ signature score).

### Prediction of intrinsic subtypes of breast cancer

The intrinsic subtypes luminal A (LumA), luminal B (LumB), HER2-enriched, normal-like and basal-like were predicted by using the PAM50 centroids in the Genefu R package 35. RMA normalized gene expression data was used.

### Intratumoral immune cell composition analysis

CIBERSORT (https://cibersort.stanford.edu/) was used to calculate immune cell subtype fractions based on the gene expression profile of each breast cancer sample 36. Gene signatures (LM22) for 22 sorted immune cell subsets were used in this study. Quantile normalization was used as recommended to remove confounding effects. We chose LM22 here because the prediction power of these signatures for corresponding immune cell subsets were well experimentally validated 36.

### Statistics

Statistics was performed by using R packages including Metafor, Survival, and Survminer. Odds-ratios (OR) for the associations of neoadjuvant response with risk scores were calculated using logistic regression. The association between disease-free survival and risk scores was analyzed using Kaplan-Meier survival curves with log-rank test and cox proportional hazards regression. Disease free survival (DFS) was defined as the time from surgery to the first confirmed relapse or metastasis. In this study distant metastasis-free survival (DMFS) was preferred to be used in survival analysis, while when the data is not available, relapse-free survival (RFS) data was used. The overall hazard ratio (HR) of a variable of interest was calculated using a random-effects model. The significance of the overall effects across multiple datasets was estimated by Z test.

All statistical analyses were two-sided and considered significant when *p* < 0.05.

## Results

### IFNα, IFNγ and TNFα pathways are up-regulated while TGFβ pathway is down-regulated in BLBC

**Figure 1:**
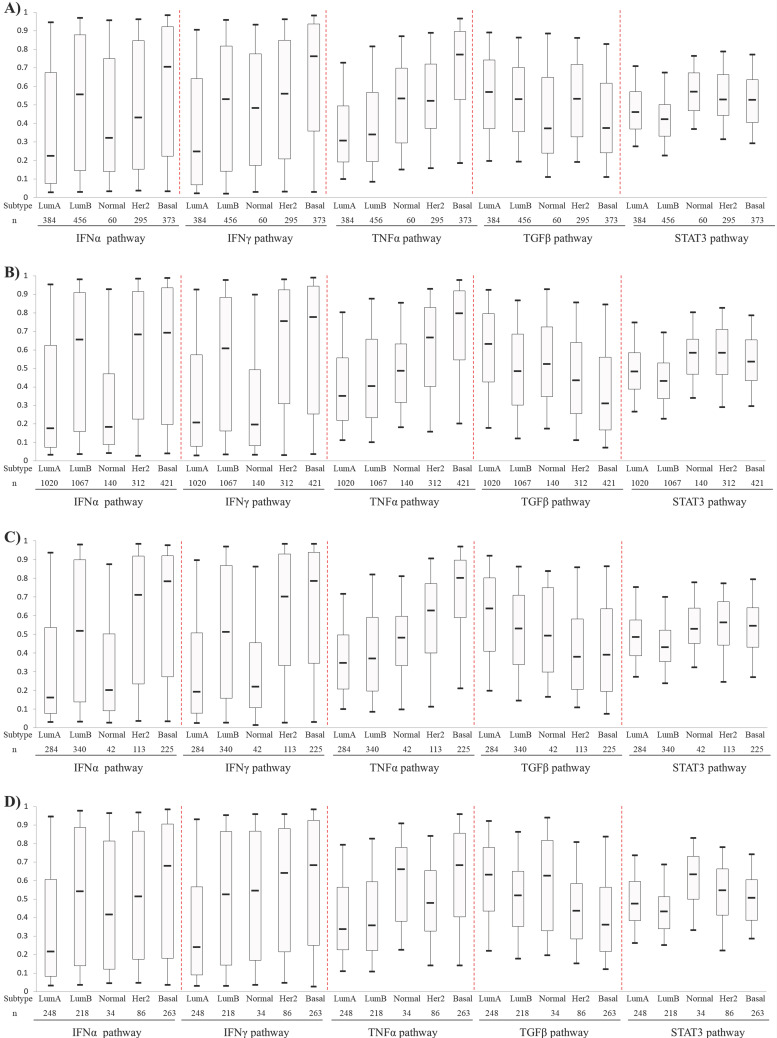
** Comparison of IFNα, IFNγ, TNFα, TGFβ and STAT3 pathway activities among PAM50-based intrinsic subtypes of breast cancers.** A) Patient cohort 1. B) Cohort 2. C) Cohort 3. D) Cohort 4. The four BC cohorts were merged from 42 Affymetrix microarray datasets as described in Materials and methods. Box-Whisker plots were used to show pathway activity, and the five statistics (5th, 25th, 50th, 75th and 95th percentile) were represented by the lower whisker, the lower box part, the solid line, the upper box part and the upper whisker, respectively. Notably, three datasets (GSE16446, GSE25055 and GSE25065) were present in both Cohort 1 and Cohort 4. To avoid repeated counting of sample data here, these three datasets were removed from cohort 1 when calculating pathway activity. Lum A: luminal A; Lum B: luminal B; Normal: Normal-like; Her2: HER2-enriched; Basal: basal-like.

Activities of the IFNα, IFNγ, TNFα TGFβ and STAT3 pathways, which were predicted with corresponding signatures, were compared among five PAM50-based BC subtypes. The first four pathways showed different expression pattern among different BC subtypes (Fig. **1**). The IFNα and IFNγ pathways showed higher levels in HER2-enriched BCs and BLBCs but lower levels in luminal A and B BCs. The TNFα pathway activity increased gradually from luminal A, luminal B, Normal-like, HER2-enriched and basal-like subtypes. Conversely, the TGFβ pathway exhibited an opposite activity pattern with TNFα pathway. Overall, the pathway activities of IFNα, IFNγ, TNFα were the highest, while that of TGFβ was the lowest in BLBC. No significant difference of the STAT3 pathway activity was observed among the five PAM50 subtypes (Fig. **1**).

### IFNα, IFNγ, TNFα and TGFβ signaling are associated with recurrence risk of BLBC

**Figure 2:**
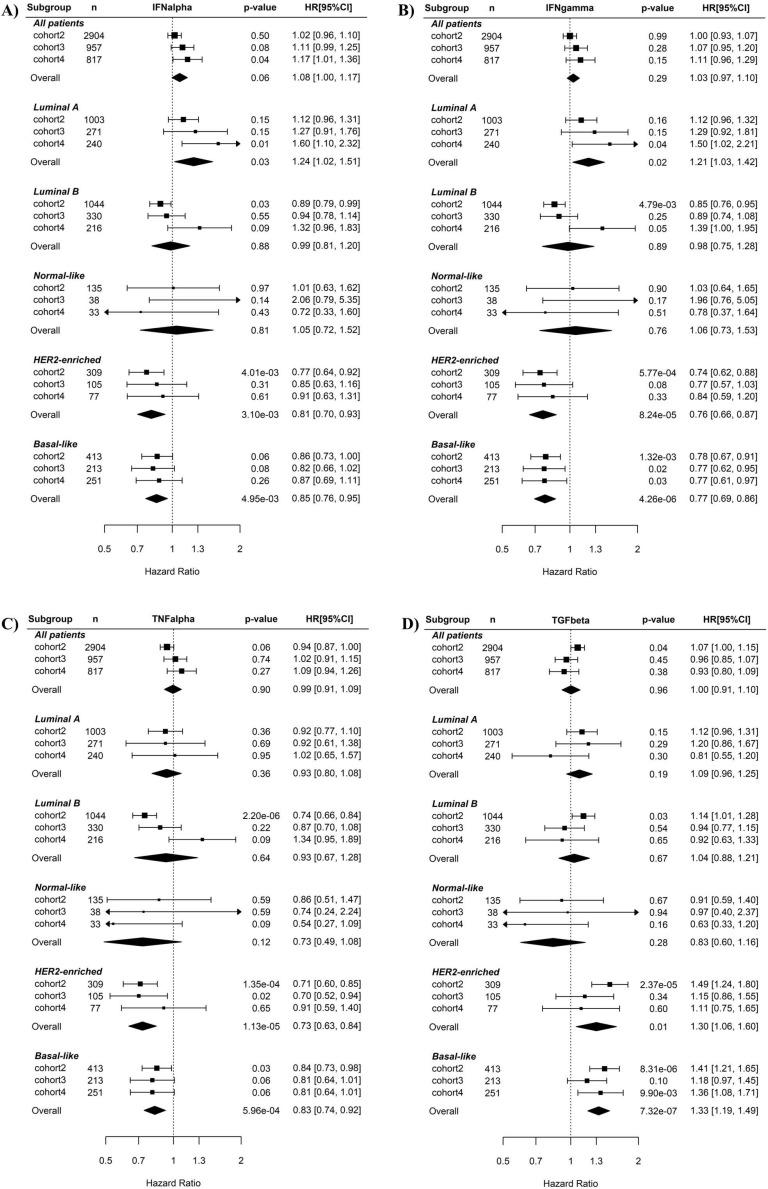
** Cox regression analysis of the associations of the IFNα, IFNγ, TNFα and TGFβ pathways with recurrence risk in different subtypes of breast cancers.** A) IFNα pathway. B) IFNγ pathway. C) TNFα pathway. D) TGFβ pathway. The three BC cohorts annotated with patient's survival information were analyzed here. The pathway activities were used as continuous variables. The recurrence risk with the increase of the pathway activity was indicated by HR (presented per one-SD increment) as shown in forest plot. The overall effect of HR was calculated using a random-effects model, and the significance of the overall effects across multiple cohorts was estimated by Z test. HRs are shown in forest plots, in which the squares and horizontal lines represent the HR and 95% CI for the individual variables, while the diamonds represent the HR and 95% CI for the overall estimate. IFNa: IFNα; IFNg: IFNγ; TNFa: TNFα; TGFb: TGFβ.

Associations of the five immunity-related pathways with BC recurrence risk were first examined using univariate Cox regression (Fig. **2**). When all BC samples were tested or just luminal A, luminal B, Normal-like BC subtypes were tested, none of the five pathways were prognostic for BC recurrence. By contrast, when basal-like subtype was tested in Cox regression, the IFNγ (overall HR 0.77, 95% CI 0.69-0.86, *p* = 4.26E-06, Fig. **2B**) and TNFα (overall HR 0.83, 95% CI 0.74-0.92, *p* = 5.96E-04, Fig. **2C**) pathways were significant favorable factors while the TGFβ pathway (overall HR 1.33, 95% CI 1.2-1.48, *p* = 1.85E-07, Fig. **2D**) was significant unfavorable factors for disease-free survival. The IFNα pathway was also a prognostic factor (overall HR 0.85, 95% CI 0.76-0.95, p = 4.95E-03, Fig. **2A**) but had weaker effect than the IFNγ and TNFα pathways. Notably, higher IFNγ and TNFα pathway activity were also associated with lower recurrence risk in HER2-enriched BC. No association between the STAT3 pathway and BC recurrence was observed in any of BC subtypes (Supplementary Fig. **S2**).

Multivariate regression analysis revealed that the association of IFNα, IFNγ, TNFα and TGFβ pathways with recurrence risk remain significant in BLBC after adjusting for different clinical variables including age, tumor grade and size, and status of lymph node, estrogen receptor (ER), progesterone receptor (PR) and HER2 in the three cohorts (Supplementary Fig. **S3**).

**Figure 3:**
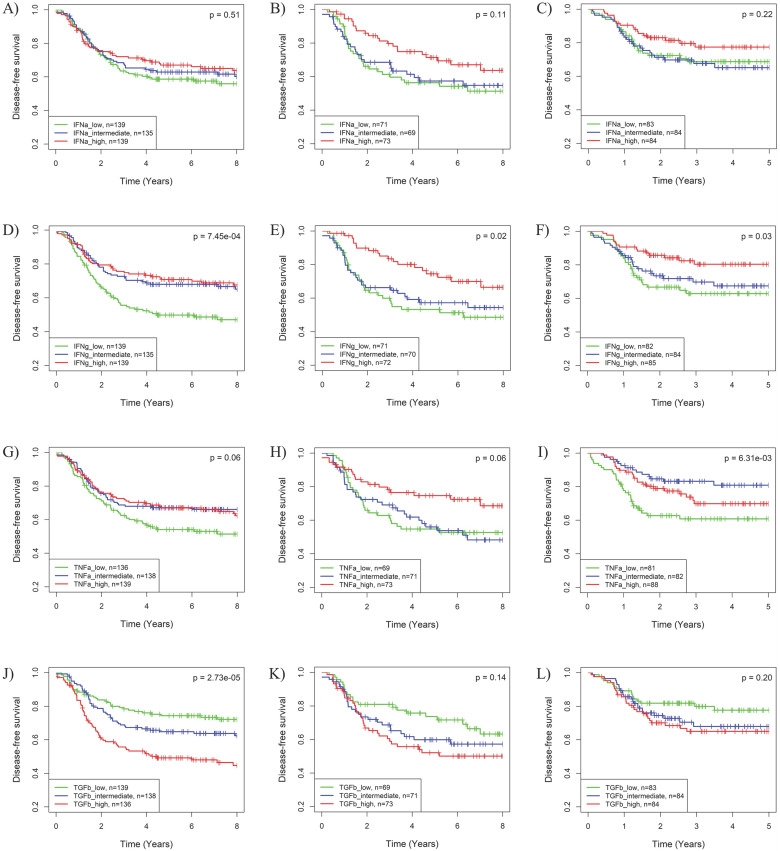
** Kaplan-Meier analysis of the associations of the IFNα, IFNγ, TNFα and TGFβ pathway activities with recurrence risk of BLBC.** The three BC cohorts annotated with patient's survival information were analyzed, including cohort 2 (A, D, G, J), cohort 3 (B, E, H, K) and cohort 4 (C, F, I, L). The BLBCs were stratified into three subgroups based on tertile splits of predicted activities of the IFNα (A-C), IFNγ (D-F), TNFα (G-I) or TGFβ (J-L) pathway (high: > 2/3 percentile; low ≤ 1/3 percentile; intermediate: ≤ 2/3 percentile and >1/3 percentile) for each cohort, and Kaplan-Meier analysis was performed to compare the probabilities of DFS among each of the three subgroups. IFNa: IFNα; IFNg: IFNγ; TNFa: TNFα; TGFb: TGFβ.

The impact of the IFNα, IFNγ, TNFα and TGFβ pathways on BLBC recurrence was further supported by Kaplan-Meier analysis. As shown in Fig. **S4,** HER2-enriched and basal-like BCs have worst prognosis among the five PAM50 subtypes in the 3 cohorts. Based on IFNγ, TNFα or TGFβ pathway activities, BLBC could be further stratified into 3 subgroups with significantly different recurrence risks (Fig. **3**). Similar to the results in Cox regression analysis, subgroups of BLBC with higher IFNγ (log-rank *p* = 7.45E-04, 0.02 and 0.03 respectively) and TNFα (log-rank *p* = 0.06, 0.06 and 6.31E-03 respectively) activity had significantly lower recurrence risk in all 3 BC cohorts (Fig. **3**). Recurrence risk of BLBC was also low in subgroups with high IFNα (log-rank *p* = 0.51, 0.11 and 0.22 respectively) or low TGFβ (log-rank *p* = 2.73E-05, 0.14 and 0.20 respectively) pathway activities, which, however, was not as significant as that for IFNγ and TNFα pathways (Fig. **3**).

### The combined application of TNFα and TGFβ pathway activities improves the prediction of BLBC recurrence

**Figure 4:**
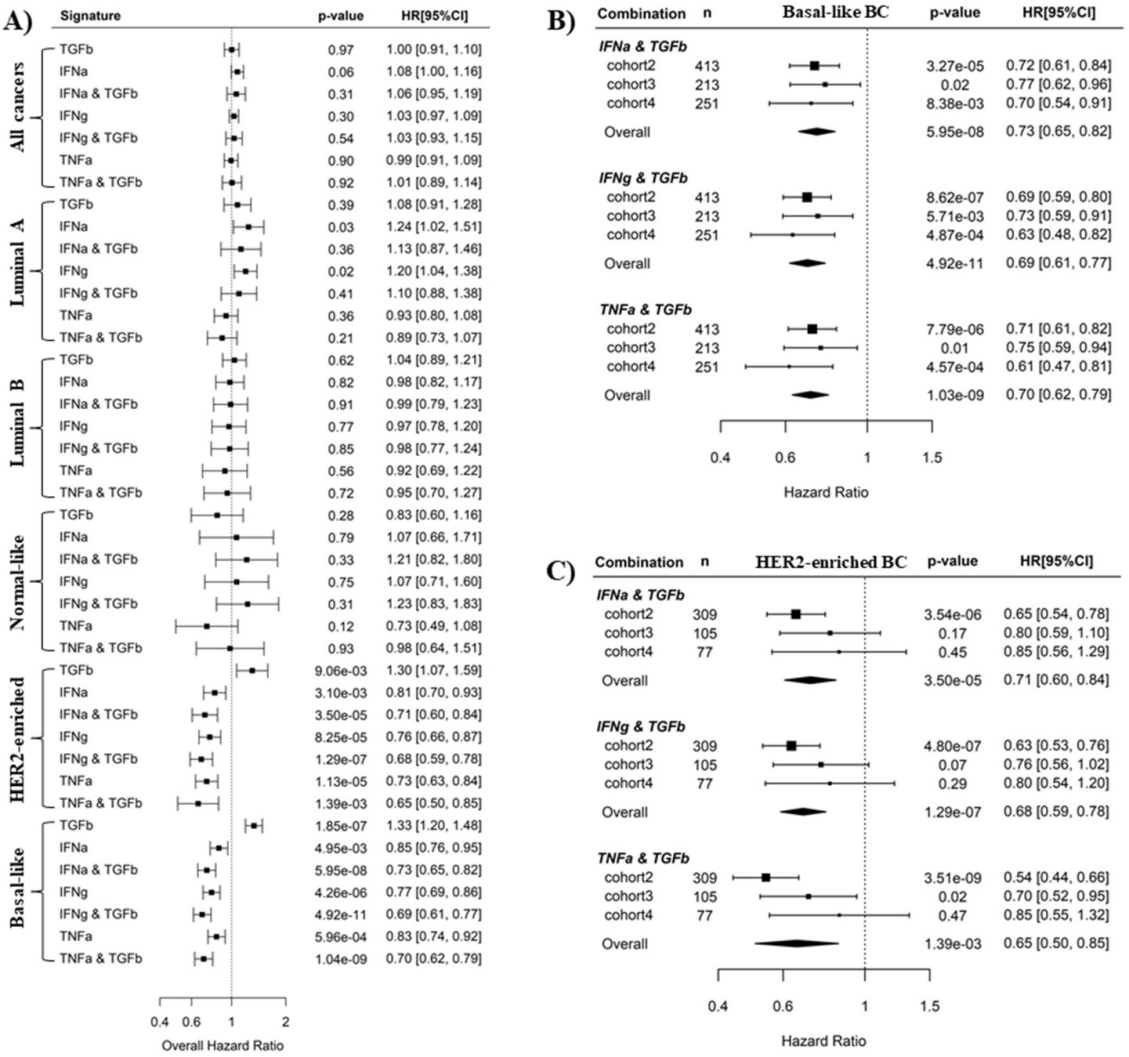
** Pathway combinations improve the prediction of BLBC prognosis.** A). Comparison of prediction efficiency of various pathway combinations in five PAM50 subtypes BC. Combination of the TGFβ pathway with the IFNα, IFNγ or TNFα pathways was tested here as indicated. The overall HR for recurrence risk shown in forest plot was based on Cox regression analysis of three breast cancer cohorts as described in Methods. Scores for pathway combinations, as described in Methods, were used as continuous variable in Cox regression analysis and the HR [95% CI] is presented per one-SD increment. B) Forest plot showing the prediction efficiency of pathway combinations for BLBC in three individual cohorts. C) Forest plot showing the prediction efficiency of pathway combinations for HER2-enriched BC in three individual cohorts.

It is well-known that IFNα, IFNγ and TNFα signaling mediate antitumor immunity whereas TGFβ signaling induces tumor immunosuppression 37-42. This is consistent with our results here that IFNα, IFNγ and TNFα pathways are favorable survival factors, while TGFβ is an unfavorable survival factor in BLBC. We wondered whether the prediction of BC recurrence could be further improved by using a combination of an immunosuppressive pathway and an immune activation pathway. Pathway combinations were tested in all PAM50 subtypes, and Cox regression analysis showed that synergistic effects of pathway combination on recurrence prediction were only observed in BLBC and HER2-enriched BC (Fig. **4A**). In BLBC, the combination of TGFβ pathway with either IFNα, IFNγ or TNFα pathways archived more significant overall HR (HR = 0.73, 0.69, 0.70, and p = 5.95E-08, 4.92E-11, 1.04E-09 for the 3 combinations, respectively) with respect to the association with cancer recurrence than the 4 individual pathways (HR = 1.23, 0.85, 0.77, 0.83, and *p* = 1.85E-07, 4.95E-03, 4.26E-06, 5.96E-04, respectively) (Fig. **4A**). Moreover, the synergistic effects of these 3 combinations in recurrence prediction for BLBC were observed in all the 3 individual cohorts (Fig. **4B** and Fig. **2**). By contrast, although combination of TGFβ pathway with IFNα or IFNγ also archived more significant overall HR than individual pathways in HER2-enriched BC (Fig. **4A**), we found the combination effects were only present in cohort 2 but not in the other 2 cohorts (Fig. **4C**, Fig. **2A** and **B**).

**Figure 5:**
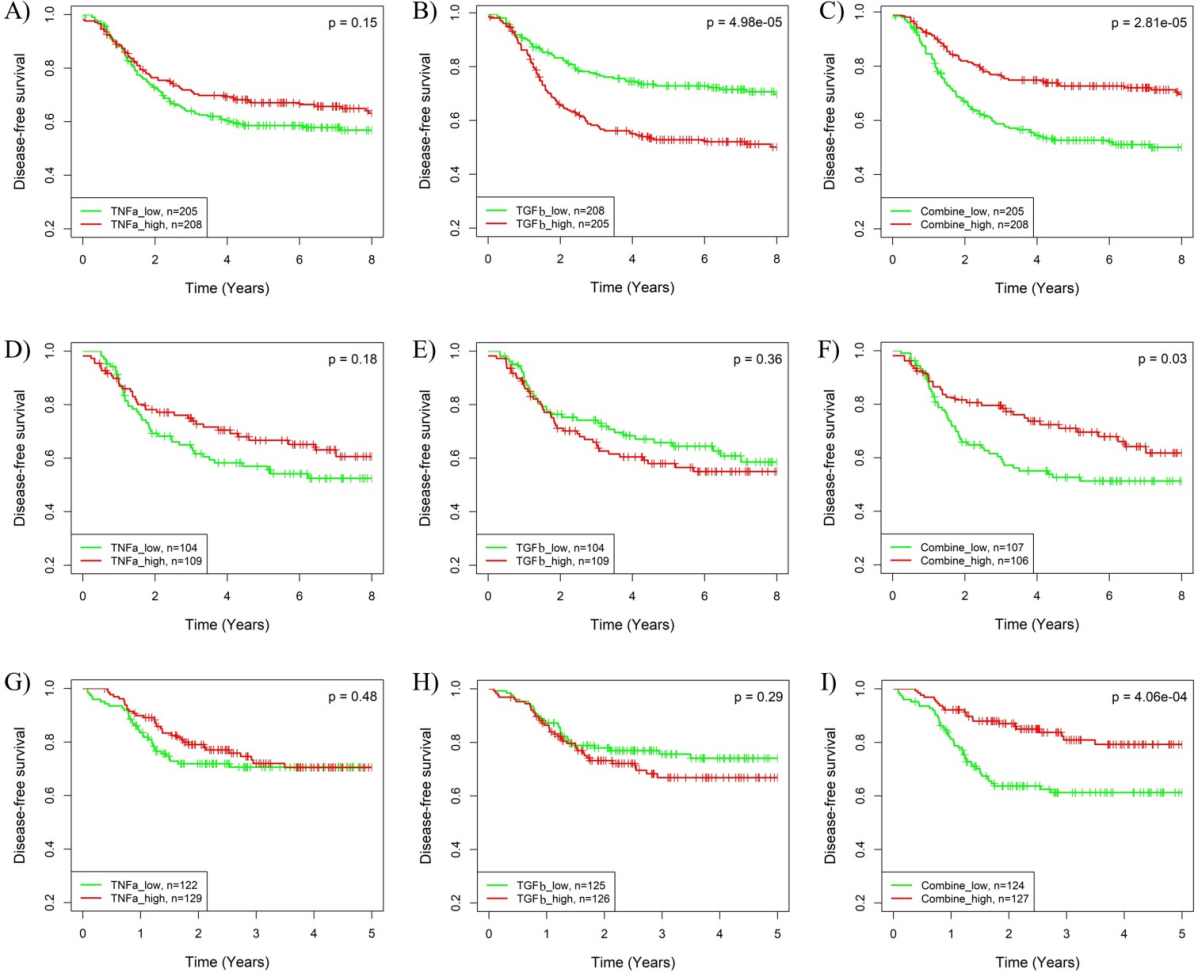
** Kaplan-Meier analysis of the synergistic effect of TNFα and TGFβ pathway in prognosis prediction of BLBCs.** Three BC cohorts with survival information were analyzed, including cohort 2 (A-C), cohort 3 (D-F) and cohort 4 (G-I). The patients were stratified into two groups based on median splits of predicted pathway activities or pathway combination scores in each cohort. A, D, G) Stratification based on TNFα pathway activity. B, E, H) Stratification based on TGFβ pathway activity. C, F, I) Stratification based on the combination scores of TNFα and TGFβ pathway. TNFa: TNFα; TGFb: TGFβ.

The synergistic effects of pathway combinations in recurrence prediction for BLBC were also evaluated using Kaplan-Meier analysis. The difference of recurrence risk among the 3 subgroups stratified based on the TGFβ & TNFα combination score (Supplementary Fig. **S5A-C**) was more significant than those stratified based on only TGFβ or TNFα pathway activity alone (Fig. **3G-L**) in all the 3 cohorts. When BLBCs were stratified into 2 subgroups, the recurrence risks were much more different among subgroups stratified based on TGFβ & TNFα combination score than those based on TGFβ or TNFα pathway alone (log-rank *p* = 2.81E-05, 0.03 and 4.06E-04 for combination *vs. p* = 4.98E-05, 0.36 and 0.29 for TGFβ alone and *p* = 0.15, 0.18 and 0.48 for TNFα alone) in the 3 cohorts (Fig. **5**). By contrast, the synergistic effect of TGFβ pathway combined with IFNα or IFNγ pathway was only observed in BLBCs from cohorts 3 and 4, regardless of whether the samples were stratified into three subgroups (Supplementary Fig. S5D-L, Fig. 3A-F) or two subgroups (Supplementary Fig. S6).

### Combined use of TNFα and TGFβ pathway signatures improve recurrence prediction for BLBC treated with adjuvant chemotherapy

Chemotherapy is the main method for treating BLBC. We asked whether these phenomena observed above are the effects of these immune-related pathway signaling on response of BC cells to chemotherapy. Analysis of BCs in cohort 1 revealed that basal-like and HER2-enriched BCs had best response to neoadjuvant chemotherapy among the 5 subtypes (Supplementary Fig. **S7A**). Cox regression analysis on BLBC in this cohort showed that none of the 4 pathways (IFNα, IFNγ, TNFα and TGFβ) were associated with neoadjuvant response (Supplementary Fig. **S7B**). Combined application of TNFα and TGFβ signatures moderately enhanced the correlation with neoadjuvant therapy responses but did not reach statistical significance (p=0.11) (Supplementary Fig. **S7B**).

We also examined the impact of the above 4 pathways on the recurrence risk of BLBC treated with adjuvant chemotherapy. Only Cohorts 2 and 4 were used in this analysis because only 21 basal-like samples in Cohort 3 had chemotherapy information annotated. Cox regression analysis revealed that only TGFβ pathway was significantly associated with recurrence of BLBC after chemotherapy treatment (Supplementary Fig. **S7C**). Combination of TGFβ with IFNγ or TNFα strongly enhanced this association in cohort 2 (Supplementary Fig. **S7C**). In Kaplan-Meier analysis, however, TGFβ and TNFα pathway combination exhibited synergistic effects on enhancing the association in both cohorts (log-rank *p* = 1.09E-03, 2.56E-02 for combination *vs. p* = 0.13, 0.63 for TNFα and* p* = 1.33E-02, 3.15E-02 for TGFβ alone and alone) (Supplementary Fig. **S8**). In contrast, in Kaplan-Meier analysis, such synergistic effects were observed only in cohort 2 for TGFβ-IFNγ pathway combination (Supplementary Fig. **S9**).

### Activated memory CD4 T cells are enriched in BLBC with high TNFα and low TGFβ pathway activity

**Figure 6:**
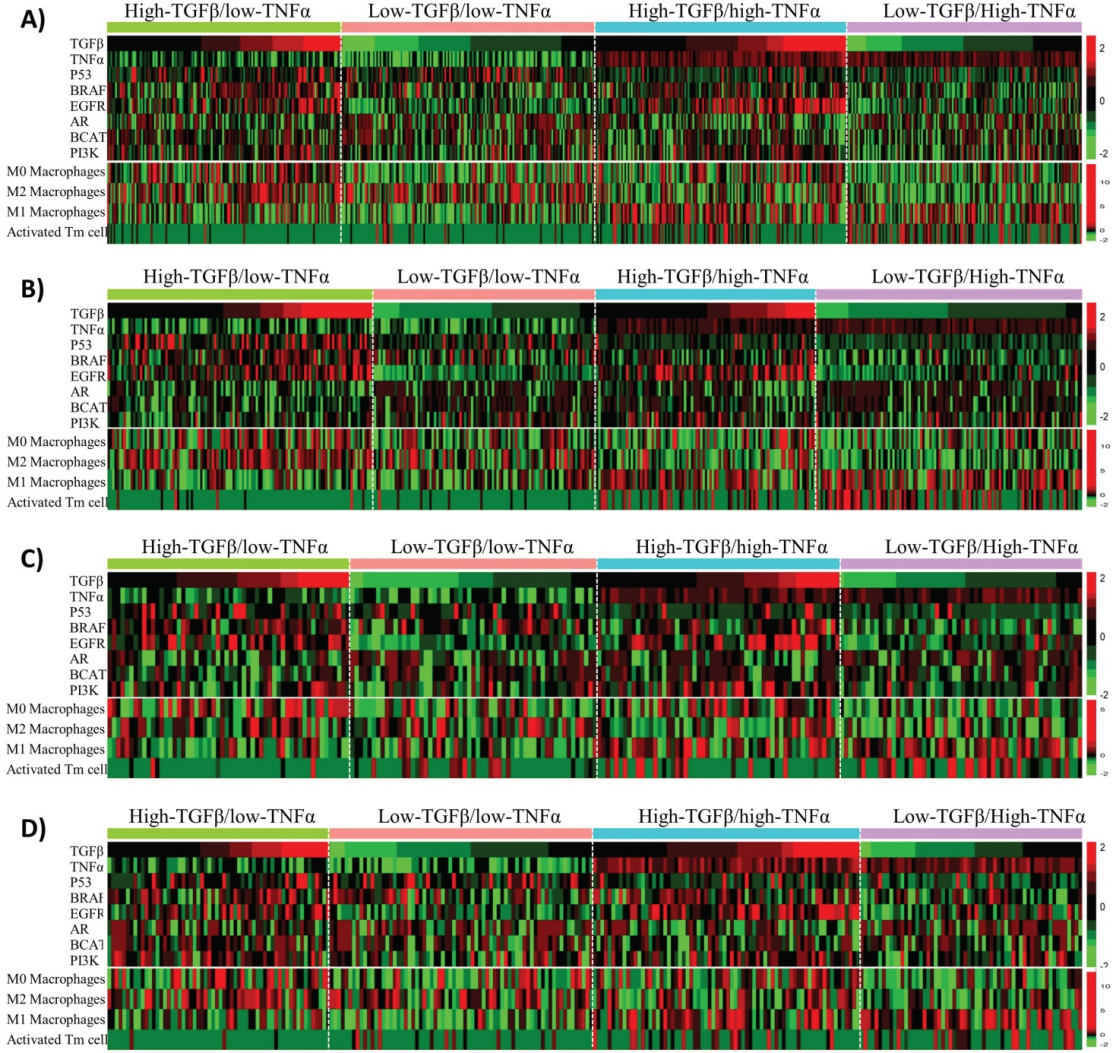
** Associations of TNFα and TGFβ pathway status with activities of six BLBC-related pathways and infiltrations of four immune cell subsets in BLBC. BLBC from four BC patient cohorts were tested.** A) Cohort 1. B) Cohort 2. C) Cohort 3. D) Cohort 4. Four BLBC subgroups, including high-TNFα/low-TGFβ, low-TNFα/high-TGFβ, high-TNFα/high-TGFβ and low-TNFα/low-TGFβ as indicated, were stratified from each cohort based on median values of the TNFα and TGFβ pathway activities, the heatmap was used to depict relative pathway activities and immune cell subset levels across the four BLBC subgroups. Each row represents one pathway or an immune cell subset as indicated. Each column represents one BLBC sample.

Besides affecting anti-cancer immunity, TNFα and TGFβ signaling also have direct tumor-suppressing or oncogenic effects on tumor cells. To uncover the potential molecular mechanisms that could explain the synergistic effects of TNFα/TGFβ pathway combination in prognostic prediction of BLBC, we first examined the associations of TNFα and TGFβ signaling with the alterations of six cancer-related pathways, including p53, BRAF, EGFR, androgen receptor (AR), beta-catenin (BCAT) and PI3K pathways that were reported to play important roles in the molecular pathogenesis of BLBC 43, 44. The BLBC were stratified into 4 subgroups in each cohort based on median values of the TNFα and TGFβ pathway activities, i.e., high-TNFα/low-TGFβ (TNFα+TGFβ-), low-TNFα/high-TGFβ (TNFα-TGFβ+), high-TNFα/high-TGFβ (TNFα+TGFβ+) and low-TNFα/low-TGFβ (TNFα-TGFβ-). We first compared the activities of the above-mentioned 6 pathways among these 4 subgroups. Enrichment of some of the pathways was associated with either TNFα or TGFβ signaling, such as p53 signaling enriched in TNFα- BC and EGFR signaling enriched in TGFβ+ BC (Fig. **6**). However, none of the 6 pathways showed specific enrichment or suppression in TNFα+TGFβ- or TNFα-TGFβ+ subgroups of BLBC (Fig. **6**).

Considering that the TNFα and TGFβ signaling strength in cancer cells reflects the TNFα and TGFβ levels in intercellular matrix, we ask whether intracellular TNFα and TGFβ signaling is associated with infiltration of any specific type of immune cells in BLBC. CIBERSORT 36 was used here to calculate the fractions of 22 immune cell subsets in BLBC. Among these 22 cell subsets, infiltrations of 4 subsets, including activated memory CD4 T (Tm) cells and resting (M0), pro-inflammatory (M1) and anti-inflammatory (M2) macrophages, were found to correlate with TNFα or TGFβ signaling in BLBC (Fig. 6, Supplementary Fig. S10). Higher TNFα signaling in cancer cells was associated with higher activated Tm cell level and lower M2/higher M1 macrophage levels, while higher TGFβ signaling was associated with higher M0 macrophages level (Fig. **6**). However, only activated Tm cells in these 4 cell subsets showed specific enrichment in BLBCs with TNFα+TGFβ- or TNFα-TGFβ+ (Fig. **6**). Activated Tm cell infiltration was mainly observed in TNFα+ particularly TNFα+TGFβ- BLBC (Fig. **6**), and this trend was more clearly presented when using a scatter plot to visualize the corresponding data shown in the heatmap (supplementary Fig. **S11**). TNFα-TGFβ- BLBC had higher level of activated Tm subset than TNFα-TGFβ+ BLBC (*p* = 0.04, Mann-Whitney U Test), while TNFα+TGFβ+ BLBC had higher level of activated Tm cells than TNFα-TGFβ- BLBC (*p* < 1E-04) and TNFα+TGFβ- BLBC had higher level than TNFα+TGFβ+ BLBC (*p* = 1E-04), suggesting that both TNFα and TGFβ signaling affect the infiltration of activated Tm cells in BLBC (supplementary Fig. **S11**).

## Discussion

At present, few clinical variables show predictive ability for prognosis of BLBC. Hence, we tried to identify genomic predictors of prognosis in patients with BLBCs. In the present study, we use four large independent breast cancer cohorts to examine the association of five immunity-related pathways with breast cancer prognosis. Analysis of these cohorts showed that among the five PAM50 subtypes of BC, BLBC and HER2-enriched BC were most sensitive to neoadjuvant chemotherapy and have worst prognosis. These results are well consistent with previous reports 45, 46, suggesting the reliability of gene expression data of these cohorts. Notably, four immunity-related pathways, including the IFNα, IFNγ, TNFα and TGFβ pathways, are associated with BLBC recurrence rate in varying degrees. While the TGFβ pathway is an unfavorable prognostic factor, the other three are favorable. Combined use of TNFα and TGFβ pathway activities improve prediction of recurrence risk of BLBC.

Here, we demonstrate that the combination of TNFα/TGFβ or IFNγ/TGFβ pathways can predict BLBC prognosis and activated CD4+ T cell levels. More importantly, TNFα/TGFβ combination has better synergistic effects in prognosis prediction than IFNγ/TGFβ combination. In BC, particularly in the aggressive triple-negative/basal-like subgroup, patient outcome is closely associated with the immune infiltration of tumors. While infiltration of cytotoxic T cells and assisting T-helper 1 cells are associated with good prognosis, infiltration of tumor-associated macrophages (TAMs) is linked to poor prognosis 47. Since interferon is the key cytokine to activate cytotoxic T cells and assist T helper cells, while TGF-β activates TAMs 48, 49, it is not surprising that interferon and TGF-β signaling are respectively a favorable and an unfavorable factor for BLBC prognosis in this study. TNF-α signaling also positively affects immune activation in the tumor microenvironment through a positive feedback mechanism between TNFα levels and M1 macrophage activation 50. In fact, the data here also show that higher TNFα signaling is associated with higher M1 and lower M2 macrophage levels (Fig. **6**). Although the association of IFNγ signaling as a single variable with cancer recurrence was more significant than that of TNFα signaling (Fig. **2** and **3**), the combined effects of TNFα/TGFβ signaling were more significant than combination of IFNγ/TGFβ signaling in BLBC (Fig. **4** and **5**). Interestingly, we observed that TNFα signaling strength increased gradually from luminal A, luminal B, Normal-like, HER2-enriched and basal-like subtypes, whereas the TGFβ signaling exhibited an exact opposite pattern (Fig. **1**). Probably, TNFα and TGFβ signaling are more complementary than IFNγ and TGFβ signaling in immune regulation, which may provide a potential explanation for better synergistic effects of TNFα/TGFβ combination in prediction of BLBC prognosis. Besides potential application in clinical outcome prediction, these findings may also help to understand the molecular pathogenesis of BLBCs.

Memory T cells are largely resting cells that become active after antigen stimulation. Analysis of T cell subpopulations in circulation, lymph nodes, and tumor sites in tumor-bearing mice revealed severe loss of T memory cells during tumor progression, indicating a continuous competition between cancer and T memory cells 51. Evaluation of the leukocyte composition in breast tumors indicated that activated T-memory cells are effector memory cells and a gene signature for this cell subset is associated with lower cancer recurrence 52, 53. It has been proposed that these tumor-specific memory cells can survive and maintain effective immunosurveillance with the function of long-term detection and removal of residual tumor cells 52. In addition, several studies reported that TNFα and TGFβ signaling are involved in the generation and activation of memory T cell populations 54-56. These data, together with our findings that BLBCs with low TGFβ and high TNFα pathway activities was enriched with activated Tm cells and has better prognosis, further support that cooperation of TNFα and TGFβ signaling may play an important anti-tumor role in BLBC through mechanisms at least including generation and activation of memory CD4 T cells.

There are currently no rigorously validated methods to guide the prognosis of BLBC patients. In fact, the data we provide here show that it is possible to develop such a test. Future studies will focus on extending these findings in more retrospective cohort of BLBC patients, and finally in a prospective-based clinical trial aimed at protecting low-risk BLBC patients from harmful and redundant adjuvant chemotherapy.

## Supplementary Material

Supplementary figures and tables.
