# Supplementary material for: Basal-like breast cancer with low TGFβ and high TNFα pathway activity is rich in activated memory CD4 T cells and has a good prognosis
Source: Int J Biol Sci. 2021 Jan 30;17(3):670–82. doi: 10.7150/ijbs.56128 (PMC7975701; doi:10.7150/ijbs.56128)
Supplement: Supplementary file 1 — Supplementary figures and tables. [file ijbsv17p0670s1.pdf]

Supplementary Table 1. Signature conditions for BRAF pathway activity prediction*

Probeset	Coefficient	Probeset	Coefficient	Signature parameter
208892_s_at	0.084441	209435_s_at	0.011602	Intercept: -10.669325
208891_at	0.078566	203367_at	0.011552	
204011_at	0.057058	201195_s_at	-0.01144	Burn-in for MCMC: 1000
201631_s_at	0.04849	209317_at	0.011036	
208893_s_at	0.047839	209433_s_at	0.010878	
210073_at	0.045858	209434_s_at	0.010827	Iterations for MCMC: 5000
204014_at	0.040523	211686_s_at	0.010775	
203348_s_at	0.040421	218239_s_at	0.01068	
221911_at	0.039717	221931_s_at	0.010668	
203349_s_at	0.038708	212672_at	0.010613	
221489_s_at	0.036407	211584_s_at	0.010553	
208712_at	0.035906	203612_at	0.010425	
209803_s_at	0.032737	203480_s_at	0.010233	
44783_s_at	0.032667	204159_at	-0.01021	
202431_s_at	0.03235	214805_at	0.010196	
203320_at	0.032343	206501_x_at	0.009985	
218839_at	0.031885	221020_s_at	0.009683	
204015_s_at	0.029972	209704_at	0.009606	
203394_s_at	0.029056	218047_at	-0.00959	
205193_at	0.027913	202332_at	0.009459	
206233_at	0.026701	207667_s_at	0.009346	
212558_at	0.024504	214427_at	0.009252	
216375_s_at	0.024259	210279_at	0.009011	
203395_s_at	0.023353	201437_s_at	0.008808	
201694_s_at	0.023318	218590_at	0.00878	
209884_s_at	0.021045	217053_x_at	0.008315	
202081_at	0.020672	203580_s_at	0.00829	
214721_x_at	-0.01948	217061_s_at	0.008279	
218247_s_at	0.018082	204067_at	-0.00786	
202695_s_at	0.017669	205698_s_at	-0.00776	
204973_at	0.0172	207515_s_at	0.006941	
210117_at	0.017127	212569_at	0.006585	
202770_s_at	-0.01701	222303_at	0.006281	
212501_at	0.016887	221221_s_at	-0.00564	
218062_x_at	-0.01664	218104_at	0.005571	
221986_s_at	-0.01633	222127_s_at	-0.00525	
207604_s_at	0.016288	218581_at	-0.00523	
211559_s_at	-0.01583	201394_s_at	-0.00515	
219177_at	0.015027	48825_at	-0.00489	
208152_s_at	0.014654	213137_s_at	0.004503	
201661_s_at	0.014517	205982_x_at	-0.00415	
201328_at	0.014317	218630_at	-0.00397	
219031_s_at	0.014163	219575_s_at	0.003866	
218670_at	0.013596	204678_s_at	-0.00365	
201662_s_at	0.013587	205725_at	-0.00323	
214786_at	0.013219	211215_x_at	-0.00307	
201642_at	0.012622	207525_s_at	-0.003	
216248_s_at	0.01261	222336_at	-0.00288	
201660_at	0.01219	220878_at	-0.00223	
221985_at	-0.01213	207111_at	-0.00208	

Supplementary Fig. S1. Generation and validation of gene signatures for the BRAF pathway. RMA normalized data was used for the signature generation and activity prediction for BRAF pathway. To generate the BRAF pathway signature, the gene expression data of five cancer cell lines (GSE10086) with wildtype BRAF was used as training set. Cells treated with or without MEK inhibitor were set as BRAF pathway 'off' and 'on', respectively. The gene expression data of mammary epithelial cell line MCF10a with forced expression of *HRAS* or *MEK1* (GSSE12764), and breast cancer cell line MCF-7 with forced expression of active MEK (GSE3542), were used to as test sets to validate the BRAF pathway signature. Each point in the plot represent one sample.

Supplementary Fig. S2. Cox regression analysis of the associations of the STAT3 pathway with recurrence risk in different subtypes of breast cancers. The three BC cohorts annotated with patient's survival information were analyzed here. The pathway activity was used as continuous variables. The recurrence risk with the increase of the pathway activity was indicated by HR (presented per one-SD increment) as shown in forest plot. The overall effect of HR was calculated using a random-effects model, and the significance of the overall effects across multiple cohorts was estimated by Z test.

Supplementary Fig. S3. Multivariate Cox regression analysis of the associations of the IFNα, IFNγ, TNFα and TGFβ pathways with recurrence risk in basal-like breast cancer. Cox regression analysis was performed in patient cohort 2 **(A-D),** cohort 3 **(E-H)** and cohort 4 **(I-L)** for pathways IFNα **(A, E, I)**, IFNγ **(B, F, J),** TNFα **(C, G, K)** and TGFβ**(D, H, L)**, respectively. The pathway activities were used as continuous

variables. All the other covariates, including age (≤50 vs >50), grade (grade 1&2 vs 3), size (≤2cm vs >2cm), ER (negative vs positive), PR (negative vs positive) and HER2 (negative vs positive) were used as categorical variable. The recurrence risk with the increase of the pathway activity in the three patient cohorts was indicated by HR (presented per one-SD increment) as shown in forest plot.

Supplementary Fig. S4. Survival curves for the five PAM50-based intrinsic subtypes in the BC cohorts used in this study. A) Cohort 2. B) Cohort 3. C) Cohort 4.

Supplementary Fig. S5. Kaplan-Meier analysis of the synergistic effects of pathway combination on prognosis prediction of BLBC. A-C) TGFβ and TNFα pathway combination. **D-F)** TGFβ and IFNα pathway combination. **G-I)** TGFβ and IFNγ pathway combination. Three BC cohorts with survival information were analyzed, including cohort 2 **(A, D, J)**, cohort 3 **(B, E, H)** and cohort 4 **(C, G, K)**. The patients were stratified into three subgroups based on tertile splits of the scores of corresponding pathway combination. The Kaplan-Meier analysis data for subgroups stratified based on individual pathways are shown in **Fig.2**.

Supplementary Fig. S6. Kaplan-Meier analysis of the synergistic effects of pathway combination on prognosis prediction of BLBC. A-C) Subgroups stratified into based on IFNα pathway activity. **D-F)** Subgroups stratified into based on TGFβ and IFNα pathway combination scores. **G-I)** Subgroups stratified into based on IFNγ pathway activity. **J-L)** Subgroups stratified into based on TGFβ and IFNα pathway combination scores. Three BC cohorts with survival information were analyzed, including cohort 2 **(A, D, G, J)**, cohort 3 **(B, E, H, K)** and cohort 4 **(C, F, I, L)**. The patients were

stratified into two subgroups based on median splits of the corresponding pathway activities or pathway combination scores. The Kaplan-Meier analysis data for two subgroups stratified based on TGFβ pathway activity alone are shown in **Fig.5**.

Supplementary Fig. S7. Associations of IFNα, IFNγ, TNFα and TGFβ pathways with prognostic prediction for BLBC treated with chemotherapy. A) Comparison of response rate of five PAM50-based intrinsic subtypes to neoadjuvant chemotherapy. BC cohort 1 (*n*=2093) was analyzed here as it is annotated with patient's neoadjuvant response information. Luminal A BC was used as reference and its OR set as 1. **B)** Associations of IFNα, IFNγ, TNFα and TGFβ pathways, alone or in combination, with response rate of BLBC to neoadjuvant chemotherapy in cohort 1 (*n*=584). **C)** Associations of IFNα, IFNγ, TNFα and TGFβ pathways, alone or in combination, with BLBC recurrence risk after adjuvant chemotherapy. Only BLBC from cohorts 2 (*n*=150) and 4 (*n*=84) were tested here since there are only 21 basal-like samples in cohort 3 annotated chemotherapy information. Lum A: luminal A; Lum B: luminal B; Normal: Normal-like; Her2: HER2-enriched; Basal: basal-like; IFNa: IFNα; IFNg: IFNγ; TNFa: TNFα; TGFb: TGFβ.

Supplementary Fig. S8. Synergistic effects of TNFα and TGFβ pathways on prediction of recurrence risk for BLBC after adjuvant chemotherapy. Only cohort 2 **(A-C)** and cohort 4 **(D-F)** were tested with Kaplan-Meier plot since there are only 21 basal-like samples in cohort 3 annotated chemotherapy information. The patients were stratified into two groups based on median splits of predicted pathway activities or pathway combination scores in each cohort. **A, D)** Stratification based on TNFα

pathway activity. **B, E)** Stratification based on TGFβ pathway activity. **C, G)** Stratification based on the combination scores of TNFα and TGFβ pathway.

Supplementary Fig. S9. Synergistic effects of combination of TGFβ with IFNα or IFNγ on prediction of recurrence risk for BLBC after adjuvant chemotherapy. A, B) Subgroups stratified into based on IFNα pathway activity. **C, D)** Subgroups stratified into based on TGFβ and IFNα pathway combination scores. **E, F)** Subgroups stratified into based on IFNγ pathway activity. **G, H)** Subgroups stratified into based on TGFβ and IFNα pathway combination scores. Two BC cohorts with survival information were analyzed, including cohort 2 **(A, C, E, G)** and cohort 4 **(B, D, F, H)**. The patients were stratified into two subgroups based on median splits of the corresponding pathway activities or pathway combination scores. The Kaplan-Meier analysis data for two subgroups stratified based on TGFβ pathway activity alone are shown in **Supplementary Fig. S8**.

Supplementary Fig.S10. Associations of TNFα and TGFβ pathway status with different immune cell subsets in BLBC. BLBC from four BC patient cohorts were tested. **A)** Cohort 1. **B)** Cohort 2. **C)** Cohort 3. **D)** Cohort 4. Four BLBC subgroups, including high-TNFα/low-TGFβ, low-TNFα/high-TGFβ, high-TNFα/high-TGFβ and low-TNFα/low-TGFβ as indicated, were stratified from each cohort based on median values of the TNFα and TGFβ pathway activities, the heatmap was used to depict relative immune cell subset levels across the four BLBC subgroups. Each row represents one immune cell subset. Each column represents one BLBC sample. The fractions of 22 immune cell subsets were predicted based on gene expression data. Four

of subsets that correlated with TNFα and TGFβ pathway status were shown in **Fig.6**. Data for the remaining subsets are presented here. 1: T cells CD4 naive; 2: B cells naive; 3: B cells memory; 4: Plasma cells; 5: T cells CD8; 6: T cells CD4 memory resting; 7: T cells follicular helper; 8: T cells regulatory (Tregs); 9: T cells gamma delta; 10: NK cells resting; 11: NK cells activated; 12: Monocytes; 13: Dendritic cells resting; 14: Dendritic cells activated; 15: Mast cells resting; 16: Mast cells activated; 17: Eosinophils; 18: Neutrophils.

Supplementary Fig.S11. Comparison of levels of activated memory CD4 T cells among BLBCs with different status of high TNFα and low TGFβ pathway activities. Each dot represents one sample and the red point represents mean value of activated memory CD4 T cells in one subgroup.

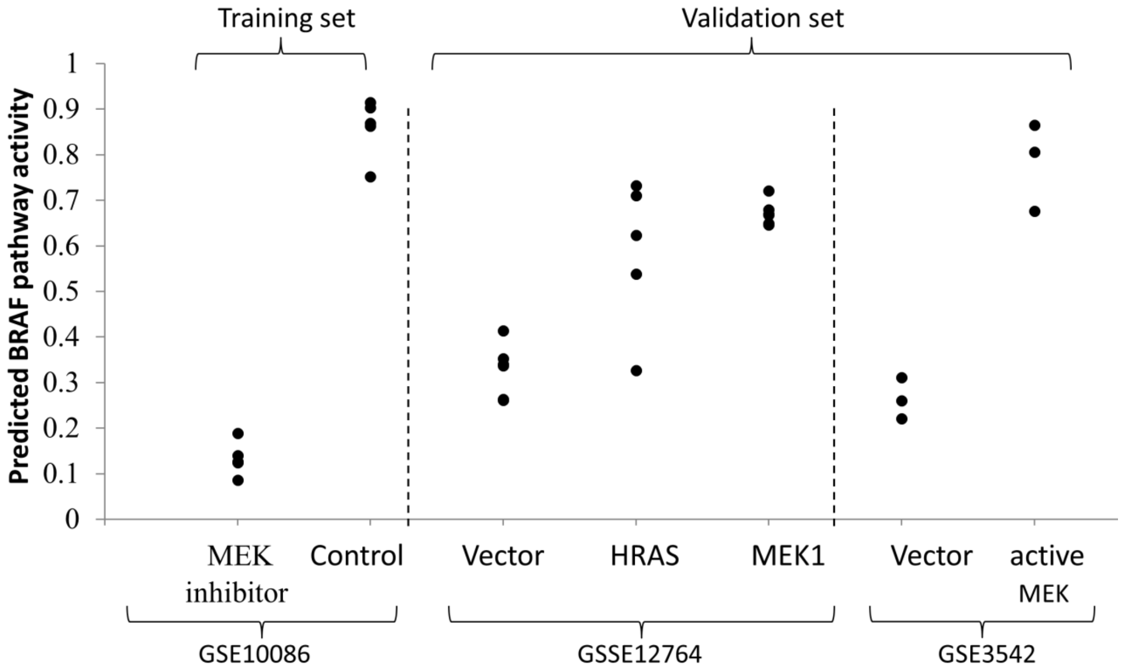

Supplementary Fig.S1

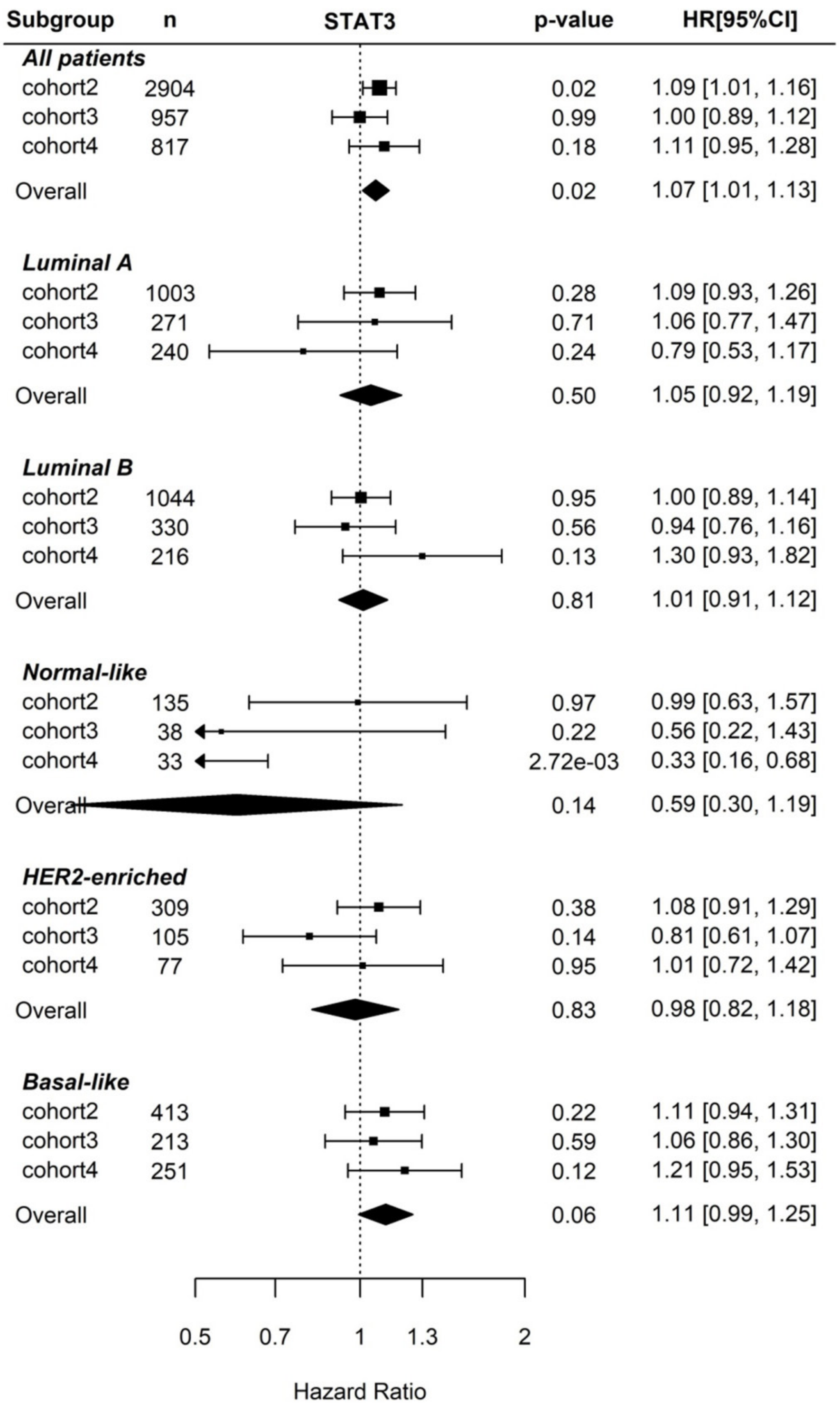

Supplementary Fig.2

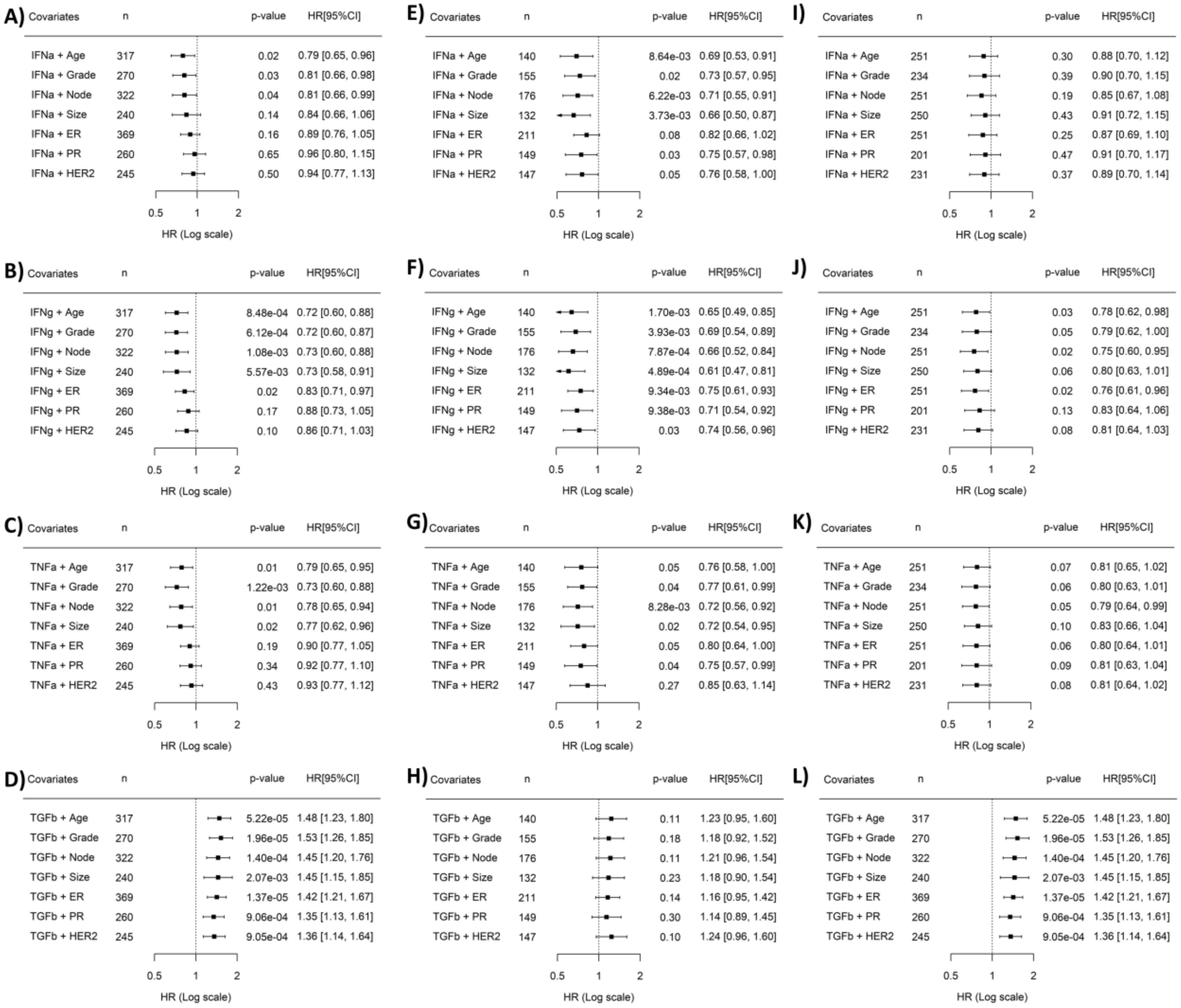

Supplementary Fig.S3

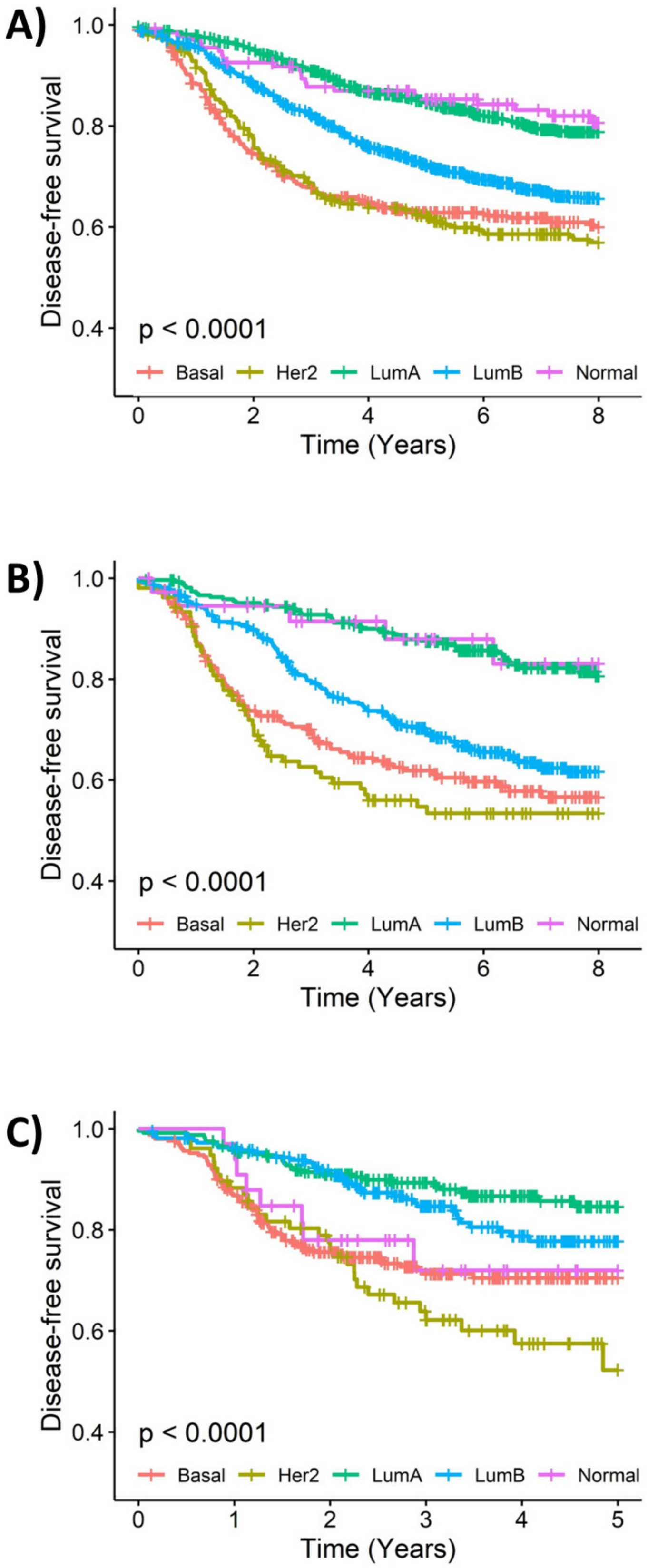

Supplementary Fig.S4

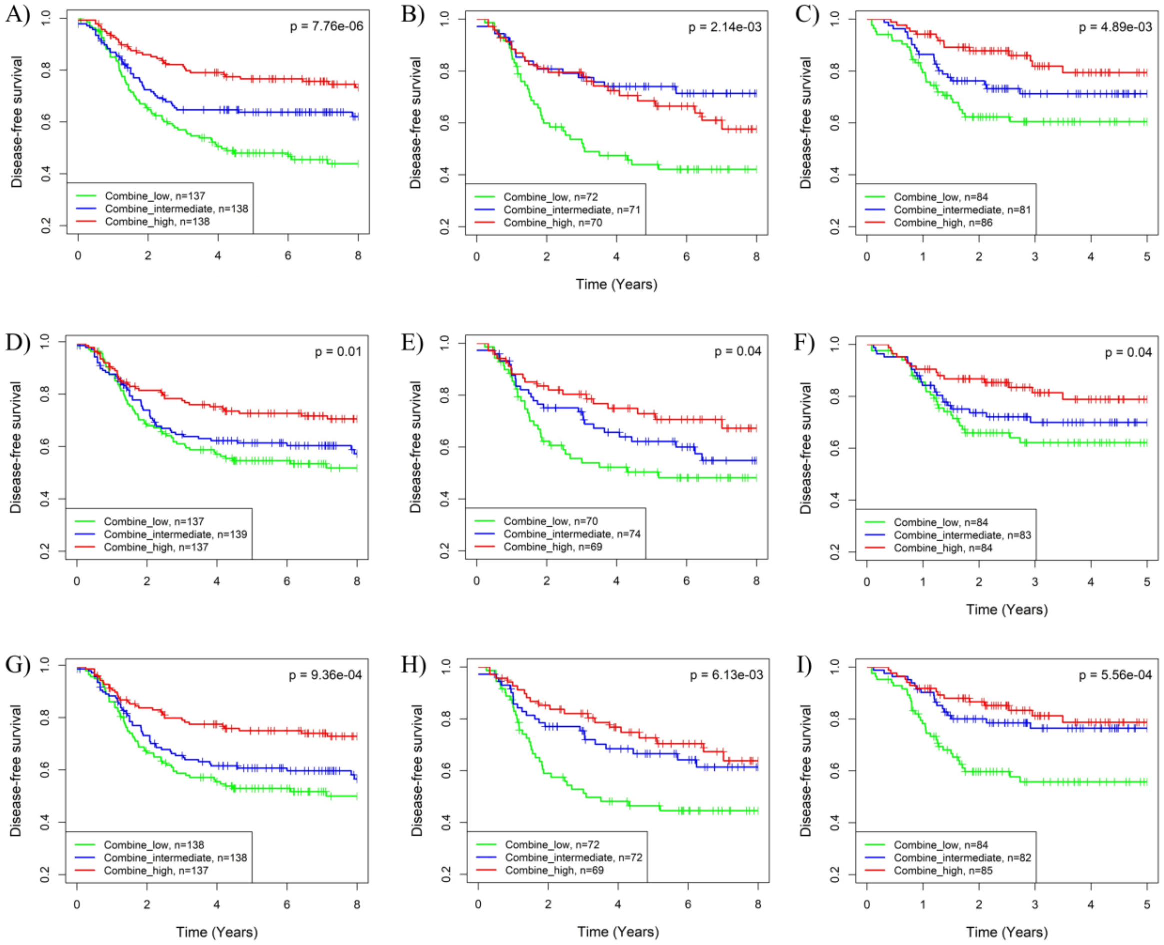

Supplementary Fig.S5

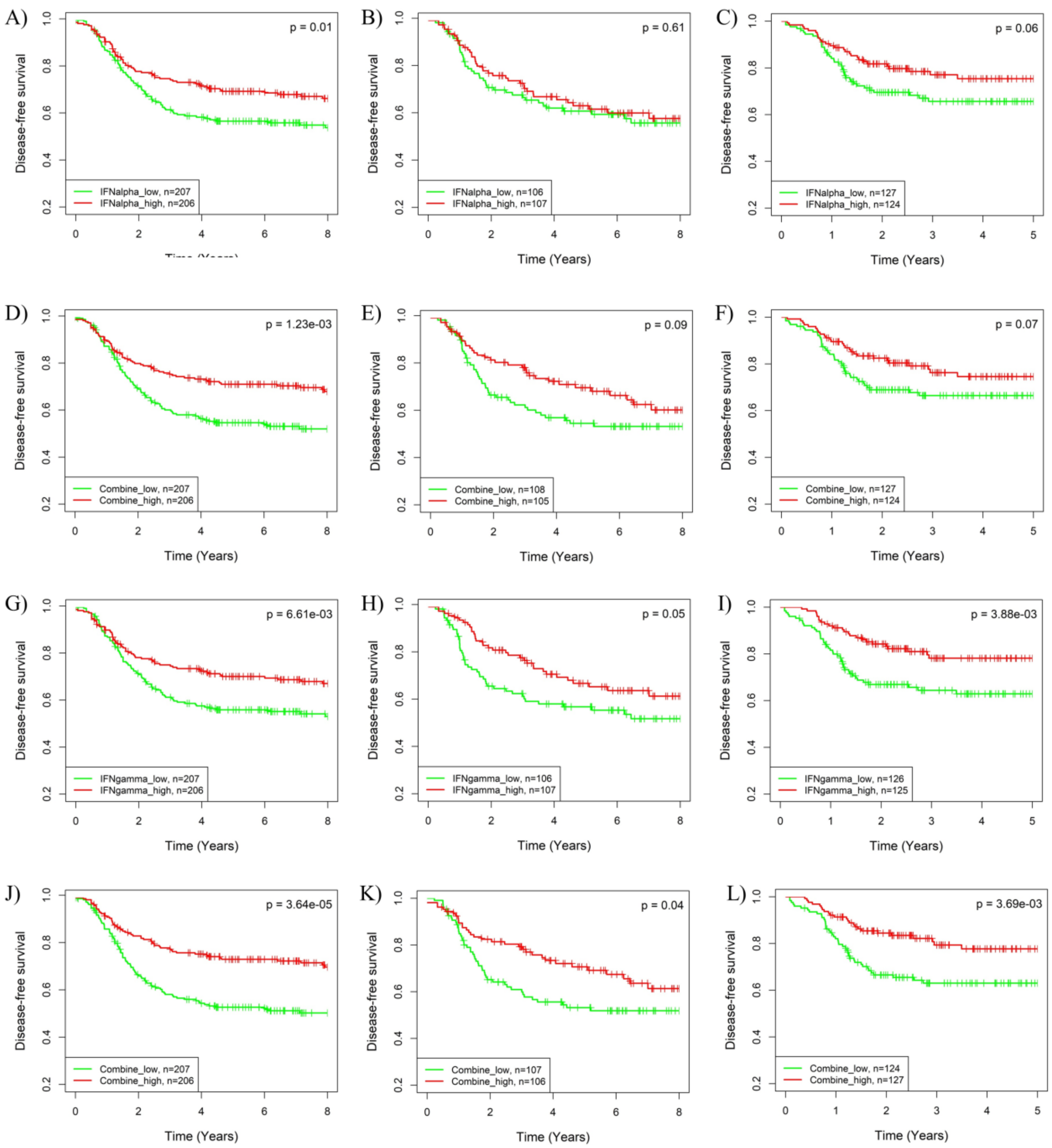

Supplementary Fig.S6

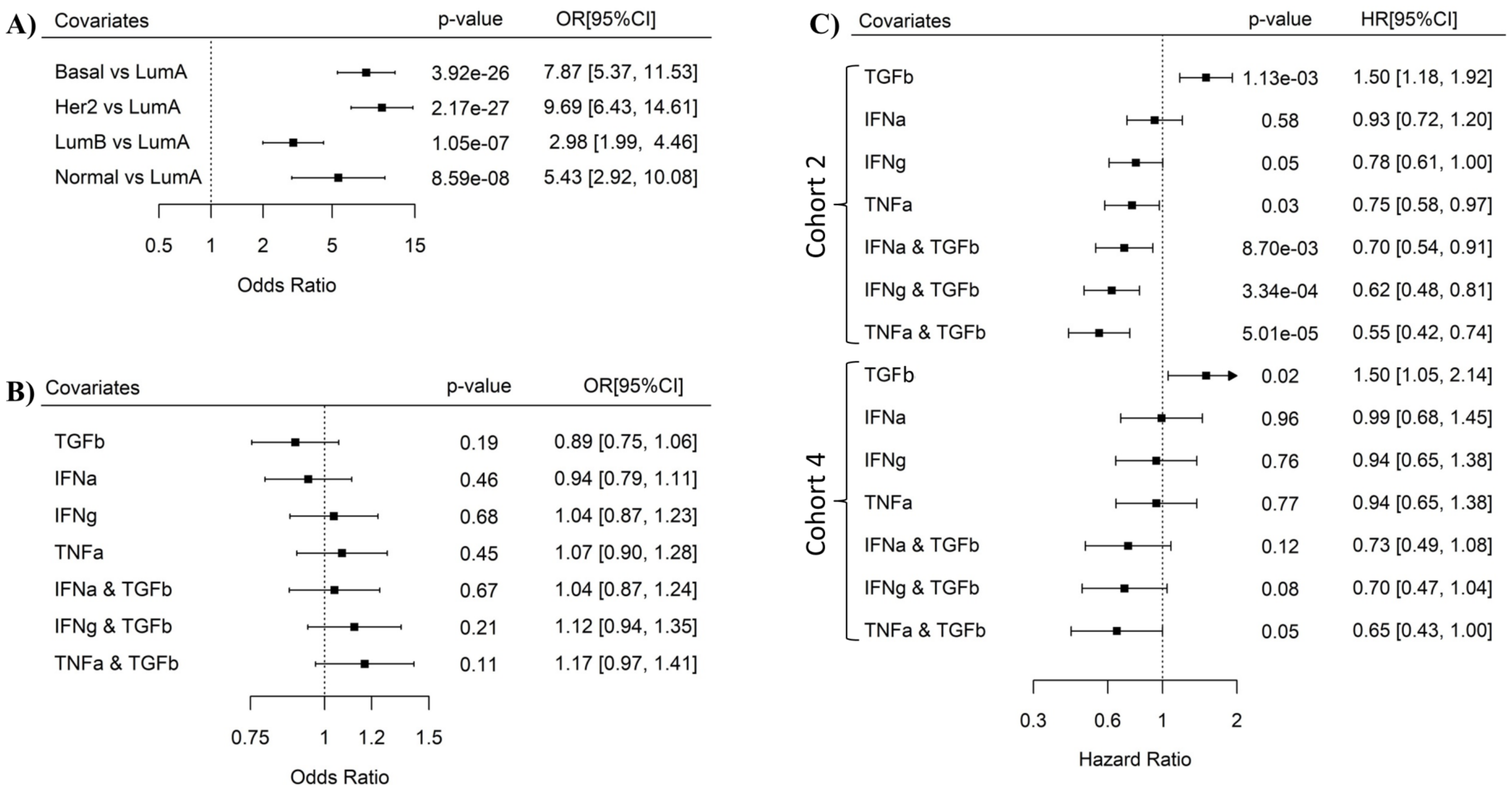

Supplementary Fig.S7

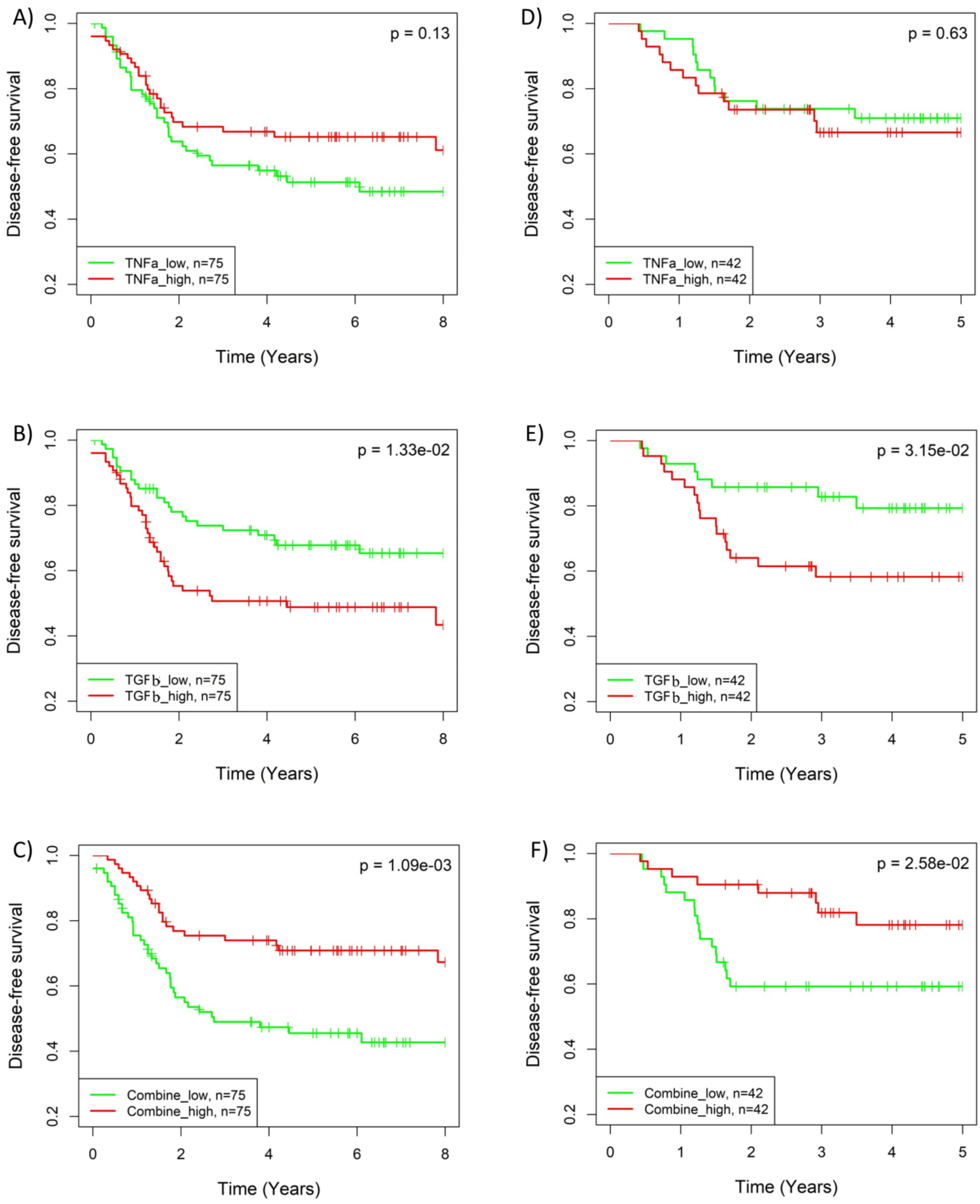

Supplementary Fig.S8

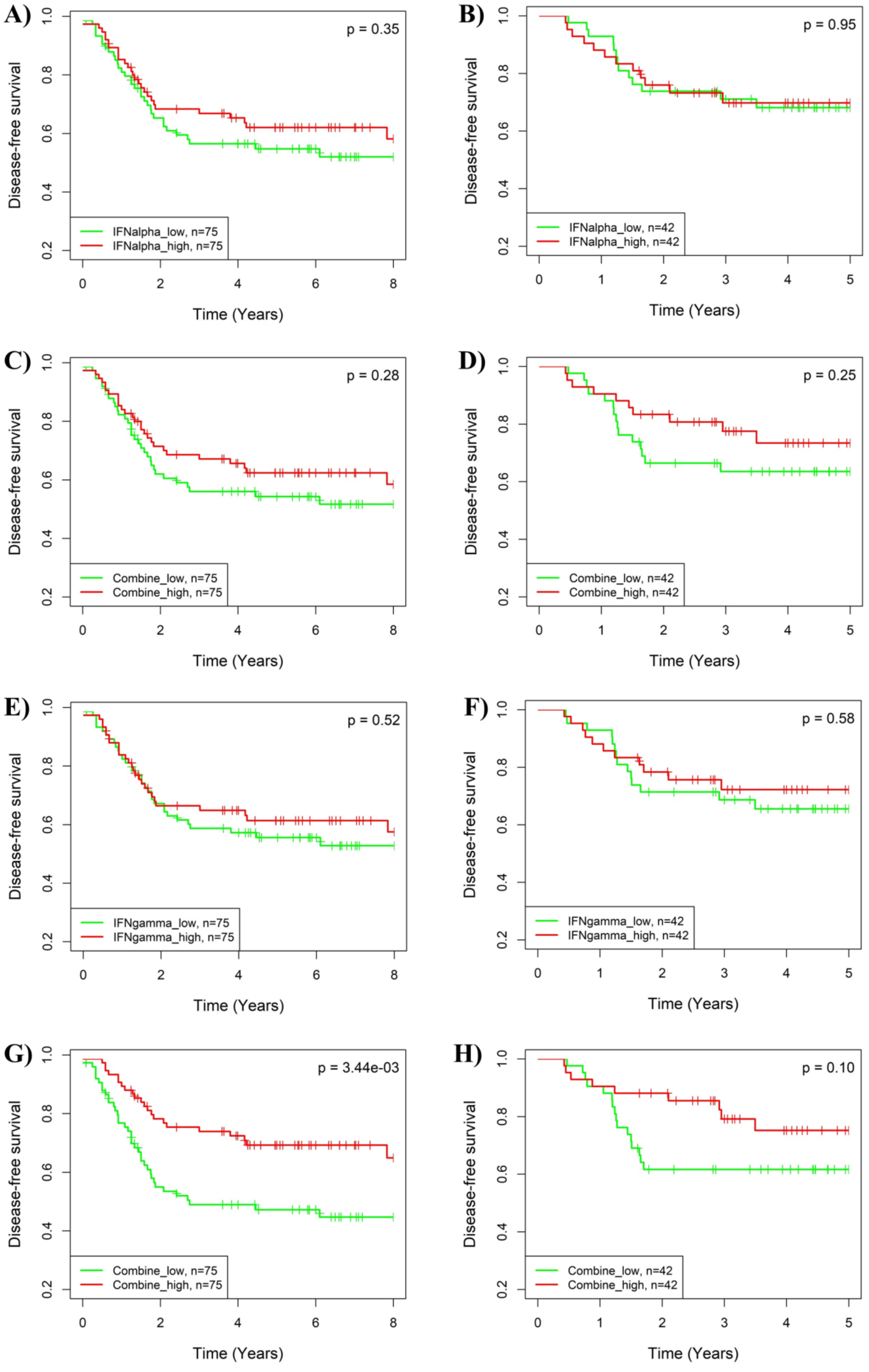

Supplementary Fig.S9

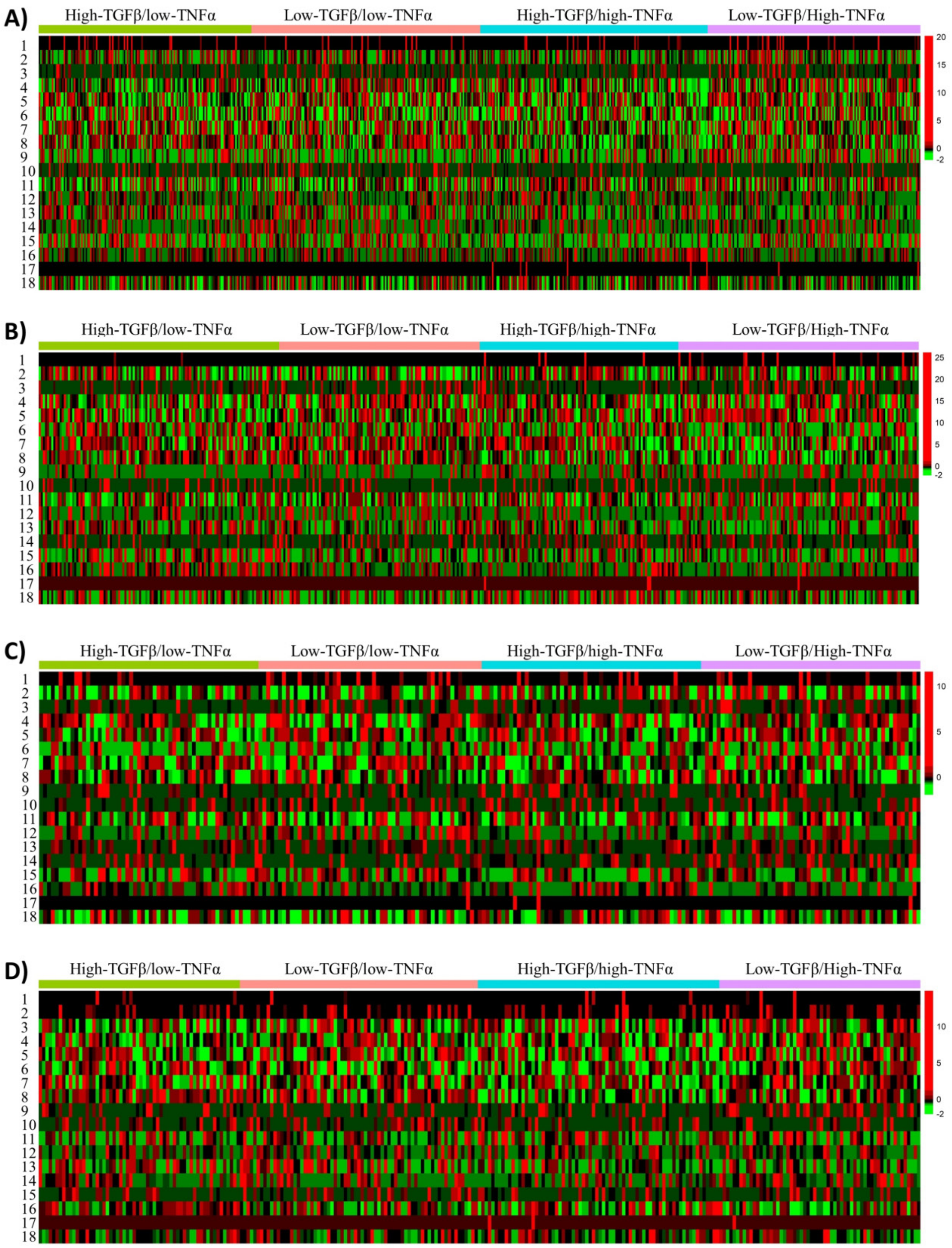

Supplementary Fig.S10

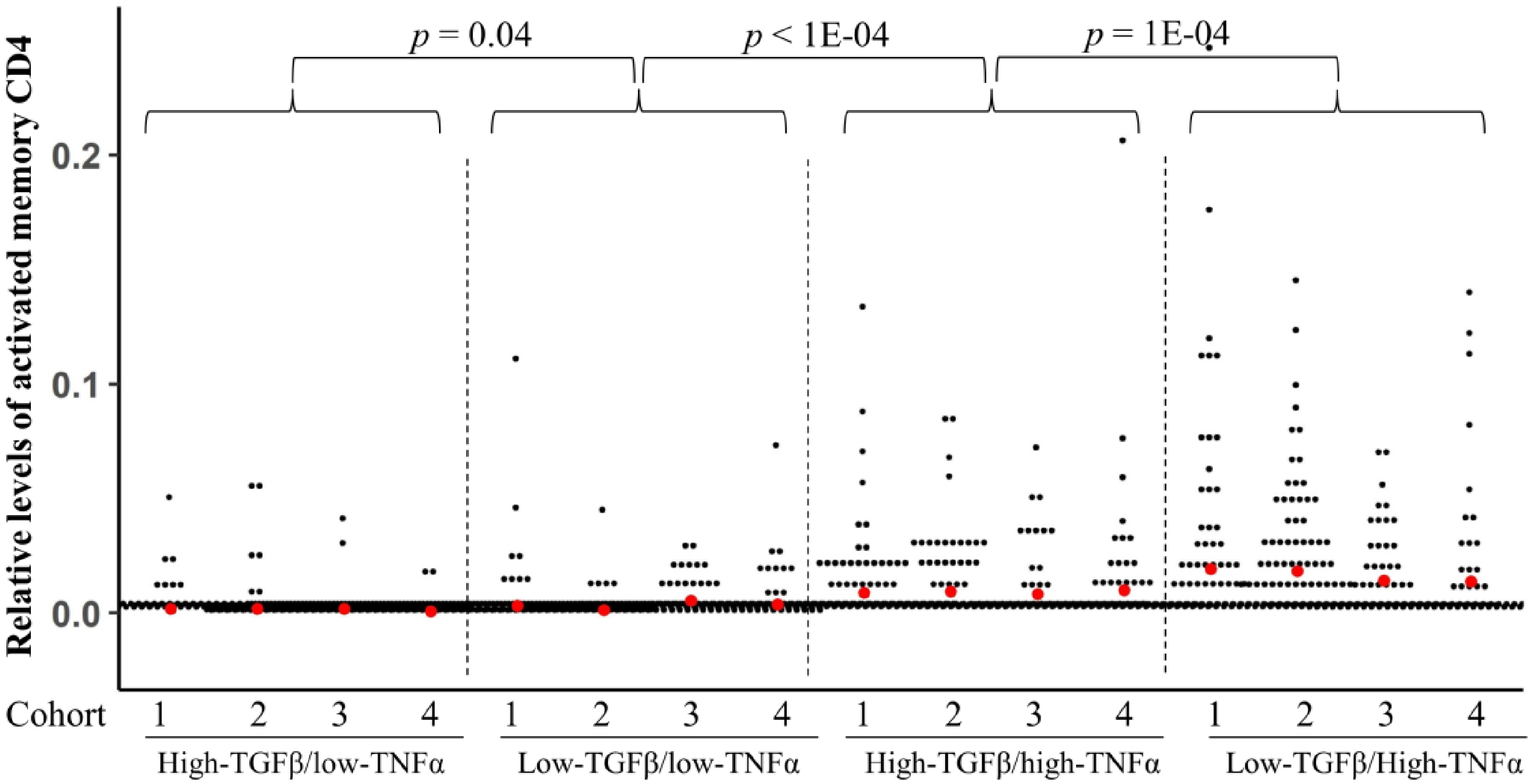

Supplementary Fig.S11
